# A Medium to Long‐Term Study Comparing Stress Urinary Incontinence Procedures

**DOI:** 10.1002/nau.70101

**Published:** 2025-07-07

**Authors:** Ifeoma Offiah, D. Carolina Ochoa, Jennifer M. Alvarado, Millie Mercer, Chendrimada Madhu, Herney A. Garcia‐Perdomo, Hashim Hashim

**Affiliations:** ^1^ Department of Obstetrics and Gynaecology North Bristol NHS Trust Bristol UK; ^2^ Bristol Urological Institute North Bristol NHS Trust Bristol UK; ^3^ Division of Urology/Urooncology, Department of Surgery, School of Medicine Universidad del Valle Cali Colombia

**Keywords:** lower urinary tract symptoms, mid‐urethral sling, patient reported outcome measures, post operative complications, stress urinary incontinence, surgery, synthetic mesh

## Abstract

**Background and Objectives:**

Concerns remain regarding the safety of the retropubic tape (TVT) procedure. We assess the efficacy, satisfaction and long‐term outcomes of the TVT procedure and compare it to the autologous fascial sling (AFS), colposuspension, and urethral bulking procedures.

**Methods:**

A review of prospective data of all patients post stress urinary incontinence (SUI) surgery in our tertiary center between January 2012 and December 2020 was performed. Patients were invited to complete three validated questionnaires: International Consultation of Incontinence Modular Questionnaire Vaginal Symptoms (ICIQ‐VS), ICIQ Female Lower Urinary Tract Symptoms (ICIQ‐FLUTS) and ICIQ satisfaction (ICIQ‐S). Fisher′s exact test was used to evaluate complications. Kruskal‐Wallis test was used to evaluate satisfaction.

**Key Findings:**

Eight hundred fifty‐three SUI procedures were performed in the study period. The responses from 395 questionnaires were analyzed. Median follow up: 5.9 years (range 1–10.1). The TVT and AFS procedures were the most efficacious procedures: 64% TVT, 62% AFS, 40% Colposuspension, 26% urethral bulking, *p* < 0.001. The AFS group had the lowest reported rate of complications: 22.6% AFS, 65.7% TVT, 67.1% colposuspension, 71.74% Urethral bulking (*p* < 0.001). Pain was the most reported complication and the AFS group had the lowest reporting of severe pain: 68% AFS, 74% TVT, 80% Colposuspension, 78% urethral bulking (*p* = 0.350).

**Limitations:**

Mesh exposure was not evaluated due to the lack of validated questionnaires.

**Conclusion:**

The AFS and TVT procedures are reported as the most successful procedures for the surgical management of SUI. Due to the associated risk of mesh complications, we recommend AFS as the procedure of choice for the surgical management of SUI.

## 1 Introduction

Stress urinary incontinence (SUI), the leakage of urine on effort, is a common condition in women causing impairment in quality of life. Since its introduction in 1995, the mid‐urethral retropubic tension‐free transvaginal tape procedure (TVT) has been a popular surgical treatment for SUI. It replaced other more invasive alternatives including the Burch Colposuspension and the autologous fascial sling (AFS). However, in the last decade, concerns regarding the use of synthetic mesh for the surgical treatment of female SUI have been raised especially in the United Kingdom (UK) through the National Health Service (NHS). This was primarily due to the risk of mesh exposure, pain, infections, and sexual dysfunction. The Cumberledge review has highlighted a lack of long‐term outcomes associated with mid‐urethral tape mesh procedures [1]. A temporary suspension of its use was introduced in the UK, while ongoing investigations and research to determine its safety profile was performed [2, 3]. There has therefore been an increased use of other procedures for the surgical management of SUI.

Recent comparative systematic reviews and meta‐analysis of randomized controlled trials have reviewed the short‐term efficacy of surgical techniques for SUI [4, 5]. Most trials report outcomes at 1 year. Other reviews which have focussed on long‐term outcomes only studied one method of SUI surgery [6]. The question of comparing long‐term efficacy between all surgical techniques for the management of SUI has not been addressed. The NHS England Mesh working group reported concerns from patients who have campaigned to raise awareness around the use of implantable devices [7]. They argue that the evidence used to justify their use is incomplete and that there are a lot more patient concerns than is immediately evident from the traditional objective research approach. For this reason, we have used patient reported outcome measures (PROMs) to subjectively quantify long‐term outcomes for the commonest four surgical procedures used for SUI.

## 2 Objectives

The primary objective was to use PROMs to compare the long‐term efficacy and complications associated with mid‐urethral sling procedures, colposuspension, AFS and urethral bulking. Secondary objectives were to evaluate the complications, overall quality of life and patient satisfaction following these surgeries.

## 3 Materials and Methods

This study had institutional approval by the local quality Governance team. A review of prospectively collected data was performed in our tertiary center for all SUI cases operated on between the years 2012, when the lower urinary tract symptoms multidisciplinary team (LUTS MDT) was set up, and 2020.

Inclusion criteria: Female patients aged 18 years or over who had surgery for Urodynamic Stress Incontinence (USI) or women with urodynamic mixed incontinence where SUI was the main symptom. Urodynamics was performed on all patients included in the study. The surgery performed was according to patient choice as per the National Institute of Health and Clinical Excellence guidance (NICE) [8].

Exclusion criteria: patients who did not respond, patients who responded but did not consent to inclusion in the study, and deceased patients.

A postal invitation pack was sent out to all patients, and a reminder was sent 1 month after. A patient information leaflet, a written informed consent and validated questionnaires were included in the pack (Appendix 1). Patients were given three validated PROMS to complete: International Consultation of Incontinence Modular Questionnaire Vaginal Symptoms (ICIQ‐VS), ICIQ Female Lower Urinary Tract Symptoms (ICIQ‐FLUTS) and ICIQ satisfaction (ICIQ‐S). The questionnaires were a series of questions numerically grading symptoms with a free text space for comments.

A power calculation was not performed as all participants who met the inclusion criteria in the study period were invited. Fisher′s exact test was used to evaluate complications between the groups. Kruskal‐Wallis test was used to evaluate satisfaction between the groups. The Chi‐Square Test of Homogeneity was performed to evaluate sampling error.

## 4 Quantitative Success Indicator

To assess which surgery had the best outcome, a statistical indicator was designed to quantify and compare the results of the different surgeries based on questionnaire responses. This index provides a quantitative and observational tool that allows for the evaluation of outcomes through the evolution of questionnaire variables, specifically in terms of efficacy, satisfaction, overall improvement, and pain. To achieve this, the index assigns weights (25% in this case) to questionnaire items based on their answers and the distribution of satisfactory responses. This weighting was multiplied by the proportion of patients expressing a satisfactory response to each question, and the scores obtained for each procedure were then summed. This resulted in a global score for each surgery reflecting efficacy, patient satisfaction, overall improvement, and pain—fundamental aspects for evaluating the success of a surgical intervention. This score is expressed on a scale of 0–1, where 0 represents the worst possible outcome and 1 the best.

The primary outcome was evaluating success that is, no leakage in each treatment group following surgery. Secondary objectives were to establish overall quality of life, patient satisfaction and long‐term complications.

## 5 Results

There were 853 SUI/USI procedures performed in the study period. Table 1 shows the breakdown of the procedures and responses received. Average age was 60.2 years, STD +/− 10.7 years. Of the 404 participants who returned the completed questionnaires, 395 were included in the final analysis: nine transobturator tape (TOT) responders were excluded as their numbers were too small for statistical analysis. Median follow up was 5.9 years (range 1–10.1 years).

**TABLE 1:** Breakdown of study participants.

Procedures	No. of patients	Deceased	Duplicates	Invited	Responded	% Response
TVT	476	11	8	457	226	49.5%
TOT	20	1	2	17	9	52.9%
Colposuspension	135	0	5	130	84	64.6%
Bulking agent	130	9	38	83	51	61.4%
AFS	92	2	0	90	34	37.8%
Total	853	23	53	777	404	52.0%

Abbreviations: AFS, Autologous Fascial Sling; TOT, Transobturator tapes; TVT, Retropubic Tension‐free vaginal tapes.

Success was measured using three indices: efficacy = Q.11a ICIQ‐FLUTS, satisfaction = Q.1 ICIQ‐S and patient impression of improvement = Q.2 ICIQ‐S. In terms of efficacy, TVT was significantly superior to colposuspension and urethral bulking (*p* < 0.001). There was no difference between TVT and AFS: 63.8% TVT and 62.1% AFS reported no SUI (*p* = 0.999). In terms of satisfaction, there was no statistical difference between the surgeries except for TVT versus urethral bulking (*p* < 0.001) (79.7% TVT, 74.19% AFS, 69.1% colposuspension, 56% urethral bulking). Analysis of the patient impression of improvement revealed the urethral bulking group being least satisfied, and AFS the most satisfied. (87.1% AFS, 75.9% TVT, 69.1% colposuspension, 45.8% urethral bulking, *p* < 0.001), (Table 2, and Supporting Information S1: Table 1).

**TABLE 2:** Satisfaction, efficacy, and patient global impression of improvement are compared between all four surgeries.

Question	Answer	Retropubic tape	Autologous fascial sling	Colposuspension	Urethral bulking	*p* value*
Q.11a ICIQ‐FLUTS	Never	63.8	62.1	40.2	25.5	< 0.001
Q.1 ICIQ‐S	Very, or somewhat successful	79.7	74.2	69.1	56	0.004
Q.2 ICIQ‐S.	Much, or a bit better	75.9	87.1	69.1	45.8	< 0.001

*Note:* Numbers presented are the percentage values. * = Chi‐Square test.

Analysis of complications revealed that pain (Q.11a ICIQ‐S) was the most reported complication in all groups. There was no statistical difference between the groups although the AFS group had the lowest reporting of severe pain (Table 3). In terms of vaginal pain (Q.2a ICIQ‐VS), the TVT group scored the highest in reporting no vaginal pain, though this was not statistically significant between the groups, *p* = 0.553. This response was mirrored in the bladder pain question 4a ICIQ‐FLUTS, where the TVT group once again scored highest in reporting no bladder pain, *p* = 0.35 (Table 3), but not statistically significant.

**TABLE 3:** Pain questions.

Question		Answer	Retropubic tape	Autologous fascial sling	Colposuspension	Urethral bulking	*p* value
Q.11a ICIQ‐S	How much pain are you experiencing now?	Severe pain	73.7%	67.7%	79.7%	78.3%	0.350*
		A little pain	24.4%	29.0%	17.7%	21.7%	
		No pain	1.8%	3.2%	2.5%	0.0%	
Q.4a ICIQ‐FLUTS	Do you have pain in your bladder?	yes	19.9%	32.3%	22.6%	27.5%	0.666^
		No	80.1%	67.7%	77.4%	72.5%	
Q.2a ICIQ‐VS	Are you aware of soreness in your vagina?	Most of the time	8.1%	15.6%	12.5%	6.0%	0.553*
		Sometimes	13.6%	12.5%	12.5%	20.0%	
		No pain	78.3%	71.9%	75.0%	74.0%	

*Note:* There was no statistical significance in pain reporting between the groups. * = Fisher′s exact test. ^ = Chi‐Square Test of Homogeneity.

Further analysis of complication revealed the AFS group had the lowest significant reported rate of complications; Q.12 ICIQ‐S: 22.6% AFS, 65.7% TVT, 67.1% colposuspension, 71.74% Urethral bulking (*p* < 0.001). In terms of sexual activity, (Q.10 ICIQ‐VS), 7% of the TVT group reported not having a sex life because of their vaginal symptoms. This was statistically significant across the groups: 6.9% TVT, 16.7% AFS, 17.9% Colposuspension, 12.8% and urethral bulking (*p* = 0.041).

Urinary urgency (Q.3a ICIQ‐FLUTS) was the most reported storage symptom: 55.6% TVT, 56.3% AFS, 60.2% Colposuspension, and 86.3% urethral bulking (*p* < 0.001). Intermittent stream, (Q.8a ICIQ‐FLUTS), was the most reported voiding dysfunction symptom. However, there was no difference between the groups: 37.7% TVT, 50% AFS, 36.3% Colposuspension, and 38.8% Urethral bulking (*p* = 0.591). Hesitancy was reported in 29.6% TVT, 44.8% AFS, 29.3% Colposuspension and 18% urethral bulking participants. There was no statistical difference in the groups: *p* = 0.09. Therefore, although not statistically significant, the AFS group reported more difficulty with voiding dysfunction than the other groups.

Sensitivity analyses limited to patients with follow‐up durations of more than 60 months demonstrated similar outcomes for TVT and AFS: efficacy 58.3% TVT versus 50% AFS (Table 4). However, there was a significant reduction in efficacy i.e. cure of SUI for all procedures in the long‐term compared with the short to medium‐term analysis (*p* < 0.001). This was mirrored with satisfaction (*p* = 0.001) and patient impression of improvement (*p* = 0.008).

**TABLE 4:** Comparison of outcomes in the medium and long term.

Question	Answer	Time	Retropubic tape %	Autologous fascial sling %	Colposuspension %	Urethral Bulking %
Q.11a ICIQ‐FLUTS	Never	< 60 months	63.9%	60%	34.8%	28.6%
		> 60 months	58.3%	50%	41%	25%
		P‐value	< 0.001	0.080	0.109	0.211
Q.1 ICIQ‐S	Very, or somewhat successful	< 60 months	78.7%	70%	82.6%	71.4%
		> 60 months	75%	66.7%	63.9%	52.3%
		P‐value	< 0.001	0.999	0.754	0.005
Q.2 ICIQ‐S	Much, or a bit better	< 60 months	75.2%%	90%	73.9%	71.4%
		> 60 months	75%	75%	67.2%	38.6%
		*p*‐value	< 0.001	< 0.001	< 0.001	0.002

*Note:* There was a decline in all three outcome measures for all groups over time.

Urinary urgency (Q.9 ICIQ‐FLUTS) improved significantly, with less women reporting urgency in the long term versus medium term (*p* = 0.026). Regarding other functional indices, although there was a trend towards improved outcomes in the long‐term, complications were comparable with the medium term (Supporting Information S1: Table 3). Concern regarding vaginal symptoms (Q.14 ICIQ‐VS) increased significantly, with more women expressing the greatest deal of bother from their vaginal symptoms in the long term versus the medium term (*p* < 0.001).

Quantitative success indicator: Overall, when all the responses of the questionnaires were appraised in combination, specifically evaluating efficacy, satisfaction, global improvement, and pain, the TVT and AFS procedures were reported to be the most successful of all four procedures, with a total score of 0.682 TVT and 0.678 AFS. This was followed by colposuspension 0.575, and finally urethral bulking 0.441 (Table 5). The TVT and AFS groups were comparable in long‐term efficacy.

**TABLE 5:** Quantitative success indicator.

Item	Efficacy (0.25)	Satisfaction (0.25)	Patient impression of improvement (0.25)	Pain questions (0.25)			Total
question	Q.11a ICIQ‐FLUTS	Q.1 ICIQ‐S	Q.2 ICIQ‐S	Q.4a ICIQ‐FLUTS	Q.11a ICIQ‐S	Q.2a ICIQ‐VS	
	0.25	0.25	0.25	0.084	0.083	0.083	1
Retropubic tape	0.64	0.80	0.76	0.80	0.02	0.78	0.682
Autologous fascial sling	0.62	0.74	0.87	0.68	0.03	0.72	0.678
Colposuspension	0.40	0.69	0.69	0.77	0.03	0.75	0.575
Urethral bulking	0.26	0.56	0.46	0.73	0.00	0.74	0.441

*Note:* The AFS group had a lower bladder and vaginal pain reporting, however the TVT group scored higher on the satisfaction scale, resulting in a higher overall composite score. The low composite score for the urethral bulking group is secondary to their low satisfaction score.

## 6 Discussion

This survey directly compares all four techniques for the surgical management of SUI. We showed using PROMs that the AFS and the TVT were the best surgical options for the management of SUI/USI. Our results mirror those found in the literature, particularly ESTER, which underscores the superior effectiveness of AFS and retropubic midurethral slings (MUS) [9]. Likewise, the SISter Trial highlights a notably higher success rate with AFS [10]. Similarly, the TOMUS trial, emphasizes successes associated with retropubic MUS [11]. With paucity of data from randomized trials comparing these four procedures, this study provides us with high quality evidence for patient care.

Both AFS and TVT outperformed colposuspension and bulking in satisfaction levels. AFS showed 87% satisfaction closely matching the SISter trial′s 83% satisfaction rate [12]. TVT had a 79% satisfaction rate, slightly lower than some studies reporting up to 86% [11]. Urethral bulking consistently ranked lowest in satisfaction, consistent with findings from a randomized controlled trial comparing urethral bulking and TVT, where satisfaction rates were 59% for urethral bulking and 95% for TVT [13].

In our study, the complication rate, defined as “any complication,” was comparatively higher than in other publications, likely influenced by the subjective nature of the evaluation for that question. Pain emerged as the most frequently reported complication. The challenge in establishing the true prevalence across studies lies in the diverse definitions and measurements of pain in individual trials and systematic reviews [14].

We used the vaginal symptoms questionnaire to evaluate sexual functioning following surgery. This questionnaire is normally used for prolapse symptom quantification. However, it is employed here as a validated questionnaire assessing vaginal symptoms. The results therefore could have the bias of prolapse symptoms confounding the postoperative symptoms. In addition, as it is impossible to determine from the questionnaire responses if prolapse symptoms were present before the surgery, we have not further analyzed this as a post operative complication.

Urinary urgency emerged as the most prevalent urinary symptom, with a significantly higher prevalence noted post urethral bulking. This finding aligns with other studies [13]. Women with both SUI and mixed urinary incontinence were recruited to this study. This we feel, explains the high incidence of urinary urgency. AFS exhibited a higher prevalence of voiding dysfunction, akin to the results seen in the SISter trial [12]. AFS traditionally was a more occlusive procedure than colposuspension or urethral bulking as it was inserted towards the bladder neck in neurogenic patients using non‐dissolvable sutures. However, the AFS technique over the years has changed. Since about 2017 we have started inserting the AFS, using a sling‐on‐a‐string technique, with dissolvable sutures which is tension‐free and mid‐urethral.

The TVT procedure was designed as a minimally invasive procedure using polypropylene mesh. Pain related to trans‐vaginal mesh procedures, and its associated co‐morbidities are the main concerns noted from the mesh groups. However, when TVT is compared to other anti‐incontinence procedures where mesh is not used, there was no difference in pain outcomes. It is therefore imperative that the TVT procedure for incontinence and vaginal mesh procedures for prolapse are distinguished from each other in future studies analyzing pain outcomes.

A strength of this study was the 52% response rate. Long term follow up studies such as this suffers from difficulties receiving responses from patients. In the United Kingdom, a 50% or higher response rate is considered excellent. The proportion of continent women in our study at the 5‐year follow‐up differs from that reported in other studies, notably showing a higher continent rate compared to findings in other studies [15]. Direct comparisons of outcomes are challenging due differing definitions of continence across studies. The ICIQ‐FLUTS questionnaire was used to assess success, defining continence as a response of “never;” Q.11a. This approach is a particular strength of our study as the questionnaire allows differentiation between all incontinence episodes arising from SUI or insensible loss. By reporting urgency urinary incontinence separately from stress urinary incontinence, we enhance the accuracy of analyzing the true percentage of cure following surgical management of SUI. As with other published literature, success for all surgical techniques reduces in the long‐term [16].

Another strength of the study was the incorporation of shared decision making in the management pathway. Following full evaluation of symptoms, urodynamic findings, and MDT discussion, all patients were carefully counseled about all four surgical options and were allowed to choose which procedure they wished to have. Patients thus benefit from understanding the care and support available to them, empowers the patients, and improves outcomes. We have previously published data on how patient choice has changed over 5 years with a reduction in the proportion of patients opting for the TVT mesh as opposed to the AFS or colposuspension [17]. Finally, the use of PROMS to assess subjective outcomes given the concerns highlighted by patients in the NHS England Mesh Working group is a strength of the study as it describes patient perspective of outcomes, thus reducing the bias of health care personnel′s impression of outcome.

A limitation of this study was the exclusion of the TOT participants. 53% of the 17 TOT participants eligible for inclusion responded. This number was too small for statistical analysis. Previous studies have demonstrated decreased efficacy and satisfaction of TOT versus TVT [18]. The most recent NICE guidelines do not recommend offering TOT except in exceptional circumstances [8]. Patients following TOT are historically reported to have a higher risk of prolonged postoperative pain, especially thigh pain, than the retropubic procedures [19, 20, 21]. Inclusion of the TOT mesh group into the analysis might have resulted in a higher percentage of patients reporting severe pain. However, as the TOT and TVT procedures are different procedures, this amalgamation was deemed inappropriate.

Another limitation is the lack of assessment for erosion/extrusion/exposure from the synthetic material used in colposuspension or mesh procedures. This is mainly due to the lack of validated questionnaires that would assess for synthetic material erosion. Development of such questionnaires is greatly needed. Finally, this was a single center study. Future studies will aim to have a multicentre approach.

## 7 Conclusion

The AFS and TVT procedures are the most efficacious procedures for the cure of SUI/USI and have the highest satisfaction and impression of improvement. Pain was the most reported complication. There is decline in efficacy in the long‐term for all four surgical techniques. As mesh related complications were not assessed in this study, we would therefore recommend the AFS procedure as the surgery of choice for SUI as it avoids mesh‐related complications seen with the TVT procedure. Careful patient counseling of outcomes is required as is a shared decision‐making process with MDT discussions.

## Author Contributions

**Ifeoma Offiah:** methodology, writing – original draft, review and editing. **D. Carolina Ochoa:** writing – review and editing. **Millie Mercer:** data collection. **Chendrimada Madhu:** writing – review and editing, supervision. **Herney A. Garcia‐Perdomo:** formal analysis. **Jennifer M. Alvarado:** validation, formal analysis, data curation. **Hashim Hashim:** methodology, writing – review and editing, supervision.

## Ethics Statement

Local institutional board ethics approval was obtained for this study.

## Consent

All patents included in this study provided written informed consent. No additional permissions were required; all articles cited were referenced.

## Conflicts of Interest

The authors declare no conflicts of interest.

## Supporting information

supmat.

supmat.

supmat.

supmat.

## Data Availability

The data that support the findings of this study are available from the corresponding author upon reasonable request.

## References

[1] https://www.immdsreview.org.uk/downloads/IMMDSReview_Web.pdf.

[2] J. R. Morling , D. A. McAllister , W. Agur , et al., “Adverse Events After First, Single, Mesh and Non‐Mesh Surgical Procedures for Stress Urinary Incontinence and Pelvic Organ Prolapse in Scotland, 1997‐2016: A Population‐Based Cohort Study,” Lancet 389, no. 10069 (2017): 629–640. 28010993 10.1016/S0140-6736(16)32572-7

[3] SCENIHR (Scientific Committee on Emerging and Newly Identified Health Risks) ., The Safety of Surgical Meshes Used in Urogynecological Surgery, 3 December 2015.

[4] A. Mostafa , C. P. Lim , L. Hopper , P. Madhuvrata , and M. Abdel‐Fattah , “Single‐Incision Mini‐Slings Versus Standard Midurethral Slings in Surgical Management of Female Stress Urinary Incontinence: An Updated Systematic Review and Meta‐Analysis of Effectiveness and Complications,” European Urology 65, no. 2 (2014): 402–427. 24055431 10.1016/j.eururo.2013.08.032

[5] G. Novara , A. Galfano , R. Boscolo‐Berto , et al., “Complication Rates of Tension‐Free Midurethral Slings in the Treatment of Female Stress Urinary Incontinence: A Systematic Review and Meta‐Analysis of Randomized Controlled Trials Comparing Tension‐Free Midurethral Tapes to Other Surgical Procedures and Different Devices,” European Urology 53, no. 2 (2008): 288–308. 18031923 10.1016/j.eururo.2007.10.073

[6] G. A. Tommaselli , C. Di Carlo , C. Formisano , A. Fabozzi , and C. Nappi , “Medium‐Term and Long‐Term Outcomes Following Placement of Midurethral Slings for Stress Urinary Incontinence: A Systematic Review and Meta‐Analysis,” International Urogynecology Journal 26 (2015): 1253–1268. 25990203 10.1007/s00192-015-2645-5

[7] N. H. S. England , Acute Care Policy and Strategy Unit. Mesh working Group Interim Report. December 2015, https://www.england.nhs.uk/ourwork/qual-clin-lead/mesh/.

[8] Urinary Incontinence and Pelvic Organ Prolapse in Women: Management (NICE guideline NG123). Published April 2019, www.nice.org.uk. 31211537

[9] M. Brazzelli , M. Javanbakht , M. Imamura , et al., “Surgical Treatments for Women With Stress Urinary Incontinence: The Ester Systematic Review and Economic Evaluation,” Health Technology Assessment 23, no. 14 (2019): 1–306. 10.3310/hta23140 PMC6462840 30929658

[10] S. Tennstedt , The Stress Incontinence Surgical Treatment Efficacy Trial (V5) [Dataset] (NIDDK Central Repository, 2024), 10.58020/ryre-f394.

[11] H. E. Richter , M. E. Albo , H. M. Zyczynski , et al., “Retropubic Versus Transobturator Midurethral Slings for Stresse Incontinence,” New England Journal of Medicine 362, no. 22 (2010): 2066–2076. 20479459 10.1056/NEJMoa0912658 PMC2962585

[12] L. Brubaker , H. E. Richter , P. A. Norton , et al., “5‐Year Continence Rates, Satisfaction and Adverse Events of Burch Urethropexy and Fascial Sling Surgery for Urinary Incontinence,” Journal of Urology 187, no. 4 (2012): 1324–1330. 22341290 10.1016/j.juro.2011.11.087 PMC3586411

[13] A. M. Itkonen Freitas , M. Mentula , P. Rahkola‐Soisalo , S. Tulokas , and T. S. Mikkola , “Tension‐Free Vaginal Tape Surgery Versus Polyacrylamide Hydrogel Injection for Primary Stress Urinary Incontinence: A Randomized Clinical Trial,” Journal of Urology 203, no. 2 (2020): 372–378. 31479396 10.1097/JU.0000000000000517

[14] A. Kuprasertkul and P. Zimmern , “Challenges of Very Long‐Term Reporting in Stress Urinary Incontinence Surgeries in Women,” Urology 139 (2020): 50–59. 32032686 10.1016/j.urology.2020.01.028

[15] F. Fusco , M. Abdel‐Fattah , C. R. Chapple , et al., “Updated Systematic Review and Meta‐Analysis of the Comparative Data on Colposuspensions, Pubovaginal Slings, and Midurethral Tapes in the Surgical Treatment of Female Stress Urinary Incontinence,” European Urology 72, no. 4 (2017): 567–591. 28479203 10.1016/j.eururo.2017.04.026

[16] I. Nygaard , L. Brubaker , H. M. Zyczynski , et al., “Long‐Term Outcomes Following Abdominal Sacrocolpopexy for Pelvic Organ Prolapse,” Journal of the American Medical Association 309, no. 19 (2013): 2016–2024. 23677313 10.1001/jama.2013.4919 PMC3747840

[17] M. Wyndaele , C. Madhu , and H. Hashim , “Female Stress Urinary Incontinence Surgery: “Resurgence of the Titans,” Turkish Journal of Urology 47, no. 3 (2021): 210–215. 35929875 10.5152/tud.2021.21073 PMC8260089

[18] I. Offiah and R. Freeman , “Long‐Term Efficacy and Complications of a Multicentre Randomised Controlled Trial Comparing Retropubic and Transobturator Mid‐Urethral Slings: A Prospective Observational Study,” BJOG: An International Journal of Obstetrics & Gynaecology 128, no. 13 (2021): 2191–2199. 34478604 10.1111/1471-0528.16899

[19] P. Latthe , R. Foon , and P. Toozs‐Hobson , “Transobturator and Retropubic Tape Procedures in Stress Urinary Incontinence: A Systematic Review and Meta‐Analysis of Effectiveness and Complications,” BJOG: An International Journal of Obstetrics & Gynaecology 114, no. 5 (2007): 522–531. 17362484 10.1111/j.1471-0528.2007.01268.x

[20] S. Ross , S. Tang , M. Eliasziw , et al., “Transobturator Tape Versus Retropubic Tension‐Free Vaginal Tape for Stress Urinary Incontinence: 5‐Year Safety and Effectiveness Outcomes Following a Randomised Trial,” International Urogynecology Journal 27, no. 6 (2016): 879–886. 26670575 10.1007/s00192-015-2902-7

[21] X. Biardeau , M. Zanaty , F. Aoun , S. Benbouzid , and B. Peyronnet , “Approach and Complications Associated With Suburethral Synthetic Slings in Women: Systematic Review and Meta‐Analysis,” Progres en urologie: journal de l′Association francaise d′urologie et de la Societe francaise d′urologie 26, no. 4 (2016): 254–269. 10.1016/j.purol.2015.08.314 26372534

