# Supplementary material for: A Medium to Long‐Term Study Comparing Stress Urinary Incontinence Procedures
Source: Neurourol Urodyn. 2025 Jul 7;44(7):1425–31. doi: 10.1002/nau.70101 (PMC12319477; doi:10.1002/nau.70101)
Supplement: Supplementary file 1 — supmat. [file NAU-44-1425-s003.docx]

Supplementary Tables:

Supplementary Table 1: TVT and AFS are compared to the other surgeries in terms of satisfaction, efficacy, and patient impression of improvement. Numbers presented are the p-values indicating group differences. AFS and TVT are equivalent in all 3 items. * = Chi-Square test.

Supplementary Table 2: Presented is the breakdown of the surgeries in comparing the long versus medium term follow up results.

Supplementary Table 3: Comparison of medium-term complications with long-term complications for all surgeries. Presented are the number of participants. Overall, there was a significant reduction in urgency urinary incontinence in the long-term, and vaginal symptoms were reported as more bothersome in the long term. * = Fisher's exact test.
