# Supplementary material for: A Medium to Long‐Term Study Comparing Stress Urinary Incontinence Procedures
Source: Neurourol Urodyn. 2025 Jul 7;44(7):1425–31. doi: 10.1002/nau.70101 (PMC12319477; doi:10.1002/nau.70101)
Supplement: Supplementary file 2 — supmat. [file NAU-44-1425-s002.pdf]

Initial number | ICIQ-FLUTS 08/04 | DAY MONTH YEAR

CONFIDENTIAL

Today's date

Urinary symptoms

Many people experience urinary symptoms some of the time. We are trying to find out how many people experience urinary symptoms, and how much they bother them. We would be grateful if you could answer the following questions, thinking about how you have been, on average, over the PAST FOUR WEEKS.

1. Please write in your date of birth: DAY MONTH YEAR

2a. During the night, how many times do you have to get up to urinate, on average?

- none ☐ 0
- one ☐ 1
- two ☐ 2
- three ☐ 3
- four or more ☐ 4

2b. How much does this bother you?
Please ring a number between 0 (not at all) and 10 (a great deal)

0 1 2 3 4 5 6 7 8 9 **10**
not at all — a great deal

3a. Do you have a sudden need to rush to the toilet to urinate?

- never ☐ 0
- occasionally ☐ 1
- sometimes ☐ 2
- most of the time ☐ 3
- all of the time ☐ 4

3b. How much does this bother you?
Please ring a number between 0 (not at all) and 10 (a great deal)

0 1 2 3 4 5 6 7 8 9 **10**
not at all — a great deal

4a. Do you have pain in your bladder?

- never ☐ 0
- occasionally ☐ 1
- sometimes ☐ 2
- most of the time ☐ 3
- all of the time ☐ 4

4b. How much does this bother you?
Please ring a number between 0 (not at all) and 10 (a great deal)

0 1 2 3 4 5 6 7 8 9 **10**
not at all — a great deal

SAMPLE

Copyright © "ICIQ Group" : the ICIQ-FLUTS is based on the BFLUTS SF

5a. How often do you pass urine during the day?

1 to 6 times	☐	0
7 to 8 times	☐	1
9 to 10 times	☐	2
11 to 12 times	☐	3
13 or more times	☐	4

5b. How much does this bother you?
Please ring a number between 0 (not at all) and 10 (a great deal)

0 1 2 3 4 5 6 7 8 9 **10**
not at all — a great deal

F score: sum scores 2a-5a ☐ ☐

6a. Is there a delay before you can start to urinate?

never	☐	0
occasionally	☐	1
sometimes	☐	2
most of the time	☐	3
all of the time	☐	4

6b. How much does this bother you?
Please ring a number between 0 (not at all) and 10 (a great deal)

0 1 2 3 4 5 6 7 8 9 **10**
not at all — a great deal

7a. Do you have to strain to <u>urinate</u>?

never	☐	0
occasionally	☐	1
sometimes	☐	2
most of the time	☐	3
all of the time	☐	4

7b. How much does this bother you?
Please ring a number between 0 (not at all) and 10 (a great deal)

0 1 2 3 4 5 6 7 8 9 **10**
not at all — a great deal

SAMPLE

Copyright © "ICIQ Group" : the ICIQ-FLUTS is based on the BFLUTS SF

8a. Do you stop and start more than once while you urinate?

never	☐	0
occasionally	☐	1
sometimes	☐	2
most of the time	☐	3
all of the time	☐	4

8b. How much does this bother you?
Please ring a number between 0 (not at all) and 10 (a great deal)

0 1 2 3 4 5 6 7 8 9 **10**
not at all — a great deal

V score: sum scores 6a+7a+8a ☐ ☐

9a. Does urine leak before you can get to the toilet?

never	☐	0
occasionally	☐	1
sometimes	☐	2
most of the time	☐	3
all of the time	☐	4

9b. How much does this bother you?
Please ring a number between 0 (not at all) and 10 (a great deal)

0 1 2 3 4 5 6 7 8 9 **10**
not at all — a great deal

10a. How often do you leak urine?

never	☐	0
once or less per week	☐	1
two to three times per week	☐	2
once per day	☐	3
several times per day	☐	4

10b. How much does this bother you?
Please ring a number between 0 (not at all) and 10 (a great deal)

0 1 2 3 4 5 6 7 8 9 **10**
not at all — a great deal

SAMPLE

Copyright © "ICIQ Group" : the ICIQ-FLUTS is based on the BFLUTS SF

11a. Does urine leak when you are physically active, exert yourself, cough or sneeze?

never	☐	0
occasionally	☐	1
sometimes	☐	2
most of the time	☐	3
all of the time	☐	4

11b. How much does this bother you?
Please ring a number between 0 (not at all) and 10 (a great deal)

0 1 2 3 4 5 6 7 8 9 **10**
not at all — a great deal

12a. Do you ever leak urine for no obvious reason and without feeling that you want to go?

never	☐	0
occasionally	☐	1
sometimes	☐	2
most of the time	☐	3
all of the time	☐	4

12b. How much does this bother you?
Please ring a number between 0 (not at all) and 10 (a great deal)

0 1 2 3 4 5 6 7 8 9 **10**
not at all — a great deal

13a. Do you leak urine when you are asleep?

never	☐	0
occasionally	☐	1
sometimes	☐	2
most of the time	☐	3
all of the time	☐	4

13b. How much does this bother you?
Please ring a number between 0 (not at all) and 10 (a great deal)

0 1 2 3 4 5 6 7 8 9 **10**
not at all — a great deal

SAMPLE

I score: sum scores9a-13a ☐ ☐

© BFLUTS-SF

Thank you very much for answering these questions.

Copyright © "ICIQ Group" : the ICIQ-FLUTS is based on the BFLUTS SF
