# Supplementary material for: A Medium to Long‐Term Study Comparing Stress Urinary Incontinence Procedures
Source: Neurourol Urodyn. 2025 Jul 7;44(7):1425–31. doi: 10.1002/nau.70101 (PMC12319477; doi:10.1002/nau.70101)
Supplement: Supplementary file 3 — supmat. [file NAU-44-1425-s001.pdf]

Study number | ICIQ-S | DAY MONTH YEAR

CONFIDENTIAL

Today's date

Satisfaction

You recently underwent treatment or surgery at the urology department. We would be grateful if you could answer the following questions, thinking about how satisfied you were with the treatment or surgery that you had. All the information you give us is confidential and will not be disclosed to your team of doctors and nurses.

1. How would you rate the outcome of your surgery?

- very successful ☐ 4
- somewhat successful ☐ 3
- neither successful nor unsuccessful ☐ 2
- a little unsuccessful ☐ 1
- very unsuccessful ☐ 0

Please add any comments you wish below

2. Compared to how you felt before your surgery, how is your condition now?

- much better ☐ 4
- a bit better ☐ 3
- about the same ☐ 2
- a bit worse ☐ 1
- much worse ☐ 0

Please add any comments you wish below

3. Would you say you have been able to return to a 'normal life' after your surgery?

- strongly agree (or I was not limited before) ☐ 3
- agree ☐ 2
- disagree ☐ 1
- strongly disagree ☐ 0

Please add any comments you wish below

SAMPLE

4. If you were in the same situation again, would you still have the surgery?

yes, definitely	☐	4
yes, probably	☐	3
not sure	☐	2
no, probably not	☐	1
no, definitely not	☐	0

Please add any comments you wish below

5. Would you recommend this surgery to friends or relatives with similar problems?

yes, definitely	☐	4
yes, probably	☐	3
not sure	☐	2
no, probably not	☐	1
no, definitely not	☐	0

Please add any comments you wish below

6. If you had to spend the rest of your life with your symptoms as they are now, how would you feel?

perfectly happy (or I no longer have any symptoms)	☐	5
somewhat happy	☐	4
mixed feelings	☐	3
somewhat unhappy	☐	2
very unhappy	☐	1
desperate	☐	0

Please add any comments you wish below

Outcome score: sum scores items 1-6

SAMPLE

7. How prepared did you feel for the surgery?

completely prepared ☐
somewhat prepared ☐
somewhat unprepared ☐
completely unprepared ☐

Please add any comments you wish below

8. How <u>satisfied</u> were you with any explanation about your surgery?

very satisfied ☐
quite satisfied ☐
quite dissatisfied ☐
very dissatisfied ☐
I did not receive any information ☐

Please add any comments you wish below

9. After the surgery, how much pain did you experience?

none ☐
a little ☐
a lot ☐
severe pain ☐

(b) **How much did this bother you?**
Please ring a number between 0 (not at all) and 10 (a great deal)

0 1 2 3 4 5 6 7 8 9 **10**
not at all — a great deal

Please add any comments you wish below

10. How satisfied were you with any treatment you had for pain?

I did not require pain treatment ☐
very satisfied ☐
quite satisfied ☐
quite dissatisfied ☐
very dissatisfied ☐

Please add any comments you wish below

SAMPLE

11. How much pain are you experiencing now?

none ☐
a little ☐
a lot ☐
severe pain ☐

(b) **How much does this bother you?**
Please ring a number between 0 (not at all) and 10 (a great deal)

0 1 2 3 4 5 6 7 8 9 **10**
not at all — a great deal

Please add any comments you wish below

12. Were there any complications or side effects with your surgery?

no complications ☐
yes, minor complications ☐
yes, major complications ☐

Please add any comments you wish below

13. Overall, has the result of your surgery been…?

better than you expected ☐
different than expected ☐
just as you expected ☐
worse than you expected ☐

Please add any comments you wish below

14. Overall, how satisfied were you with your surgery?

Please ring a number between 0 (not satisfied) and 10 (very satisfied)

0 1 2 3 4 5 6 7 8 9 **10**
not satisfied — very satisfied

SAMPLE

Thank you very much for answering these questions.

SAMPLE
