# Supplementary material for: A Medium to Long‐Term Study Comparing Stress Urinary Incontinence Procedures
Source: Neurourol Urodyn. 2025 Jul 7;44(7):1425–31. doi: 10.1002/nau.70101 (PMC12319477; doi:10.1002/nau.70101)
Supplement: Supplementary file 4 — supmat. [file NAU-44-1425-s004.pdf]

ICIQ-VS 10/05

Initial number

CONFIDENTIAL

VAGINAL SYMPTOMS QUESTIONNAIRE

Many people experience vaginal symptoms some of the time. We are trying to find out how many people experience vaginal symptoms, and how much they bother them. We would be grateful if you could answer the following questions, thinking about how you have been, on average, over the PAST FOUR WEEKS.

Please write in today's date: DAY MONTH YEAR

Please write in your date of birth: DAY MONTH YEAR

Vaginal symptoms

1a. Are you aware of dragging pain in your lower abdomen?

- never 0
- occasionally 1
- sometimes 2
- most of the time 3
- all of the time 4

1b. How much does this bother you?
Please ring a number between 0 (not at all) and 10 (a great deal)

0 1 2 3 4 5 6 7 8 9 **10**
not at all … a great deal

2a. Are you aware of soreness in your vagina?

- never 0
- occasionally 1
- sometimes 2
- most of the time 3
- all of the time 4

2b. How much does this bother you?
Please ring a number between 0 (not at all) and 10 (a great deal)

0 1 2 3 4 5 6 7 8 9 **10**
not at all … a great deal

Copyright © "ICIQ Group"

**3a.**	**Do you feel that you have reduced sensation or feeling in or around your vagina?**	
	not at all ☐	0
	a little ☐	1
	somewhat ☐	2
	a lot ☐	3
**3b.**	**How much does this bother you?**<br>*Please ring a number between 0 (not at all) and 10 (a great deal)*	
	**0** 1 2 3 4 5 6 7 8 9 **10**<br>not at all ... a great deal	

Prolapse is a common condition affecting the normal support of the pelvic organs, which results in descent or 'dropping down' of the vaginal walls and/or the pelvic organs themselves. This can include the bladder, the bowel and the womb. Symptoms are usually worse on standing up and straining (e.g. lifting, coughing or exercising) and usually better when lying down and relaxing.

Prolapse may cause a variety of problems. We are trying to find out how many people experience prolapse, and how much this bothers them. We would be grateful if you could answer the following questions, thinking about how you have been, on average, over the PAST FOUR WEEKS.

**4a.**	**Do you feel that your vagina is too loose or lax?**	
	not at all ☐	0
	a little ☐	1
	somewhat ☐	2
	a lot ☐	3
**4b.**	**How much does this bother you?**<br>*Please ring a number between 0 (not at all) and 10 (a great deal)*	
	**0** 1 2 3 4 5 6 7 8 9 **10**<br>not at all ... a great deal	

**5a.**	**Are you aware of a lump or bulge coming down in your vagina?**	
	never ☐	0
	occasionally ☐	1
	sometimes ☐	2
	most of the time ☐	3
	all of the time ☐	4
**5b.**	**How much does this bother you?**<br>*Please ring a number between 0 (not at all) and 10 (a great deal)*	
	**0** 1 2 3 4 5 6 7 8 9 **10**<br>not at all ... a great deal	

Copyright © "ICIQ Group"

6a. **Do you feel a lump or bulge come out of your vagina, so that you can feel it on the outside or see it on the outside?**

never	☐	0
occasionally	☐	1
sometimes	☐	2
most of the time	☐	3
all of the time	☐	4

6b. **How much does this bother you?**
Please ring a number between 0 (not at all) and 10 (a great deal)

0 1 2 3 4 5 6 7 8 9 **10**
not at all — a great deal

7a. **Do you feel that your vagina is too dry?**

never	☐	0
occasionally	☐	1
sometimes	☐	2
most of the time	☐	3
all of the time	☐	4

7b. **How much does this bother you?**
Please ring a number between 0 (not at all) and 10 (a great deal)

0 1 2 3 4 5 6 7 8 9 **10**
not at all — a great deal

8a. **Do you have to insert a finger into your vagina to help empty your bowels?**

never	☐	0
occasionally	☐	1
sometimes	☐	2
most of the time	☐	3
all of the time	☐	4

8b. **How much does this bother you?**
Please ring a number between 0 (not at all) and 10 (a great deal)

0 1 2 3 4 5 6 7 8 9 **10**
not at all — a great deal

9a. **Do you feel that your vagina is too tight?**

never	☐
occasionally	☐
sometimes	☐
most of the time	☐
all of the time	☐

9b. **How much does this bother you?**
Please ring a number between 0 (not at all) and 10 (a great deal)

0 1 2 3 4 5 6 7 8 9 **10**
not at all — a great deal

Copyright © "ICIQ Group"

Sexual matters

We would be grateful if you could answer the following questions, thinking about how you have been, on average, over the PAST FOUR WEEKS.

10. Do you have a sex life at present?

- yes ☐ 1
- no, because of my vaginal symptoms ☐ 0
- no, because of other reasons ☐ 2

If NO, please go to question 14

11a. Do worries about your vagina interfere with your sex life?

- not at all ☐ 0
- a little ☐ 1
- somewhat ☐ 2
- a lot ☐ 3

11b. How much does this bother you?

Please ring a number between 0 (not at all) and 10 (a great deal)

0 1 2 3 4 5 6 7 8 9 **10**

not at all — a great deal

12a. Do you feel that your relationship with your partner is affected by vaginal symptoms?

- not at all ☐ 0
- a little ☐ 1
- somewhat ☐ 2
- a lot ☐ 3

12b. How much does this bother you?

Please ring a number between 0 (not at all) and 10 (a great deal)

0 1 2 3 4 5 6 7 8 9 **10**

not at all — a great deal

13. How much do you feel that your sex life has been spoilt by vaginal symptoms?

Please ring a number between 0 (not at all) and 10 (a great deal)

0 1 2 3 4 5 6 7 8 9 **10**

not at all — a great deal

Copyright © "ICIQ Group"

Quality of life

We would be grateful if you could answer the following questions, thinking about how you have been, on average, over the PAST FOUR WEEKS.

14. Overall, how much do vaginal symptoms interfere with your everyday life?

Please ring a number between 0 (not at all) and 10 (a great deal)

**0**	1	2	3	4	5	6	7	8	9	**10**
not at all										a great deal

Thank you very much for answering these questions.

Copyright © "ICIQ Group"

VAGINAL SYMPTOMS QUESTIONNAIRE

SCORING

(This section is for administrative use only)

Patient number

Vaginal symptoms score

Vaginal symptom score = 2×(dragging pain) + 2×(soreness in vagina) + (reduced sensation) + 2×(vagina too loose) + 2×(lump felt inside) + 2×(lump seen outside) + 2×(vagina too dry) + (faecal evacuation)

symptom*	score	weighted score
Q1. 'dragging pain'		x 2 =
Q2. 'soreness in vagina'		x 2 =
Q3. 'reduced sensation'		x 1 =
Q4. 'vagina too loose'		x 2 =
Q5. 'lump felt inside'		x 2 =
Q6. 'lump seen outside'		x 2 =
Q7. 'vagina too dry'		x 2 =
Q8. 'faecal evacuation'		x 1 =
	**Total vaginal symptoms score**	

*(Note: Q9, 'vagina too tight', is primarily for detecting a potential post-treatment complication and is therefore not included in the scoring)

Sexual matters score

Sexual matters score = (sex-life spoilt) + 8×(worries about vagina interfere with sex-life) + 8×(relationship affected)

sexual matter	score	weighted score
Q11. 'worries about vagina interfere with sex-life'		x 8 =
Q12. 'relationship affected'		x 8 =
Q13. 'sex life spoilt'		x 1 =
	**Total sexual matters score**	

Quality of life score

quality of life	score
Q14. 'quality of life affected'	

Copyright © "ICIQ Group"
